# Changes in cerebellar activity and inter-hemispheric coherence accompany improved reading performance following Quadrato Motor Training

**DOI:** 10.3389/fnsys.2014.00081

**Published:** 2014-05-09

**Authors:** Tal Dotan Ben-Soussan, Keren Avirame, Joseph Glicksohn, Abraham Goldstein, Yuval Harpaz, Michal Ben-Shachar

**Affiliations:** ^1^The Leslie and Susan Gonda (Goldschmied) Multidisciplinary Brain Research Center, Bar-Ilan UniversityRamat-Gan, Israel; ^2^Research Institute for Neuroscience, Education and Didactics, Cognitive Neurophysiology Laboratory, Patrizio Paoletti FoundationAssisi, Italy; ^3^Department of Neurology, Charité - UniversitätsmedizinBerlin, Germany; ^4^Department of Criminology, Bar-Ilan UniversityRamat-Gan, Israel; ^5^Department of Psychology, Bar-Ilan UniversityRamat-Gan, Israel; ^6^Department of English, Linguistics Division, Bar-Ilan UniversityIsrael

**Keywords:** dyslexia, MEG, motor training, cerebellum, alpha power, coherence, reading

## Abstract

Dyslexia is a multifactorial reading deficit that involves multiple brain systems. Among other theories, it has been suggested that cerebellar dysfunction may be involved in dyslexia. This theory has been supported by findings from anatomical and functional imaging. A possible rationale for cerebellar involvement in dyslexia could lie in the cerebellum’s role as an oscillator, producing synchronized activity within neuronal networks including sensorimotor networks critical for reading. If these findings are causally related to dyslexia, a training regimen that enhances cerebellar oscillatory activity should improve reading performance. We examined the cognitive and neural effects of Quadrato Motor Training (QMT), a structured sensorimotor training program that involves sequencing of motor responses based on verbal commands. Twenty-two adult Hebrew readers (12 dyslexics and 10 controls) were recruited for the study. Using Magnetoencephalography (MEG), we measured changes in alpha power and coherence following QMT in a within-subject design. Reading performance was assessed pre- and post-training using a comprehensive battery of behavioral tests. Our results demonstrate improved performance on a speeded reading task following one month of intensive QMT in both the dyslexic and control groups. Dyslexic participants, but not controls, showed significant increase in cerebellar oscillatory alpha power following training. In addition, across both time points, inter-hemispheric alpha coherence was higher in the dyslexic group compared to the control group. In conclusion, the current findings suggest that the combination of motor and language training embedded in QMT increases cerebellar oscillatory activity in dyslexics and improves reading performance. These results support the hypothesis that the cerebellum plays a role in skilled reading, and begin to unravel the underlying mechanisms that mediate cerebellar contribution in cognitive and neuronal augmentation.

## Introduction

Reading is a basic ability necessary in every-day life. Failure to acquire literacy early in the schooling years may have serious consequences for an individual’s academic achievements, well-being and employment prospects. Dyslexia, which is characterized by difficulties with accurate and fluent word recognition, poor spelling and decoding abilities, is the most common learning disability, with a prevalence rate of about 10% in school-age children (Deffenbacher et al., 2004). Longitudinal studies further indicate that dyslexia is a chronic condition that persists into adulthood (Shaywitz et al., 2008).

Difficulties in learning to read are commonly thought to derive from impaired phonemic representations and phonological processing (Bradley and Bryant, 1983; Ramus, 2004). This phonological deficit has been associated with aberrant cortical responses and altered asymmetry of activity in frontal and temporal language- and reading-related areas (Ramus, 2004; Dufor et al., 2007; Gabrieli, 2009), as well as with structural and functional abnormalities of the cerebellum (Pernet et al., 2009b).

The involvement of the cerebellum in higher cognitive functions such as language was once a controversial issue (Leiner et al., 1993; Rao et al., 2001). However, much evidence has been gathered in recent years to support this view. Initially, studies in patients with cerebellar disease reported significant deficits in verbal fluency (Akshoomoff et al., 1992; Appollonio et al., 1993). Later, cerebellar involvement was found in other aspects of language, such as phonological and semantic processing (for reviews see Stoodley and Schmahmann, 2009; Stoodley and Stein, 2011). In addition, structural imaging demonstrated that lower cerebellar declive volumes are associated with impaired reading abilities, suggesting that the cerebellum may be a biomarker of dyslexia (Pernet et al., 2009b). In fact, the cerebellar and frontal differences between dyslexics and controls are the most consistent (for reviews see Pernet et al., 2009a, b). For example, Eckert et al. (2003) found that the volume of the right anterior lobe of the cerebellum significantly distinguished dyslexic from control participants, and was correlated with reading measured by a single-word reading task.

According to broader theories, dyslexia is not limited to phonological difficulties but encompasses a wide range of neurodevelopmental deficits that can be traced back to the sensorimotor systems (Stein, 2001; Galaburda et al., 2006). It follows that difficulties in phonological processing related to dyslexia are secondary to impairments in basic sensory and motor processing. Some posit an impairment at an early stage in which fast incoming sensory information is processed in the magnocellular system (Stein and Walsh, 1997), while others have suggested a fundamental deficit in the integration of rapidly successive transient signals (Tallal et al., 1996) or in the detection of regularities in sound sequences (Oganian and Ahissar, 2012). These approaches all put forward the premise that sensorimotor alterations might be the source of the core reading impairments observed in dyslexia.

Although the role of the motor system in dyslexia is still controversial, it is by no means a novel proposal that dyslexia involves a motor component. Already in the 1930’s, Orton observed abnormal clumsiness in dyslexic children. He suggested that clumsy children could have difficulties in learning complex body movements as well as movements which are necessary for speech and writing (Orton, 1937). Studies of dyslexic participants have found impaired motor performance in a variety of tasks such as speed of tapping, heel-toe placement, rapid successive finger opposition, accuracy in copying, learning and execution of motor sequence (Nicolson et al., 2001; De Kleine and Verwey, 2009). This body of evidence supports the claims regarding the functional interactions between motor control systems, language and reading (for reviews see Hickok et al., 2011; Buckner, 2013).

The importance of cerebellar oscillatory function in neuroplasticity (Swinnen, 2002; De Zeeuw et al., 2011) and its role in motor acquisition, such as bimanual skills (e.g., Andres et al., 1999), have long been acknowledged in studies related to motor learning. Since impaired motor skills were often observed in dyslexics, some researchers attributed dyslexics’ cognitive and motor deficiencies to abnormal development and functioning of the cerebellum (Nicolson et al., 1999, 2001). These findings lead to the claim that the role of the cerebellum is not limited to regulating the rate, force, rhythm, and accuracy of movements, but also the speed, capacity, consistency and appropriateness of cognitive processes (Schmahmann, 2004; Hölzel et al., 2011; Buckner, 2013).

Consequently, several training studies aimed to improve reading through integrated sensory stimulation, incorporating visuomotor and vestibular home-based exercise program lasting 6 months (Reynolds et al., 2003; Reynolds and Nicolson, 2007). In their study, the intervention group improved in a range of motor skills, such as cerebellar/vestibular and eye movement tests, as well as in the Dyslexia Screening Test, more than the control group. Although the authors could only speculate about the neural mechanisms underlying these motor and cognitive improvements, they pointed to the involvement of cerebellar function in mediating these behavioral changes. In order to improve reading and spelling in dyslexia, other studies investigated the effect of normalizing oscillatory activity on reading and spelling using neurofeedback (Breteler et al., 2010). Following 10 weeks of neurofeedback training, the intervention group showed improved spelling in contrast to the control group; however, no improvement was found in reading performance in either group. In addition, a significant increase in alpha coherence was found, which was interpreted as an indication that attentional processes account for the observed improvement in spelling, while no correlation was found between the two measures. So far, the link between training-induced changes in cerebellar alpha oscillatory activity and reading skills remained unexplored.

In the current study, we explore the possible potential interactions between sensorimotor and reading systems, and the role of the cerebellum as a mediator between them. In a preliminary attempt to understand the causal relationship between these constructs and their role in dyslexia, we examined how reading skills change as a result of a highly-structured form of sensorimotor training. We applied *Quadrato Motor Training* (QMT), a new sensorimotor whole-body training that involves following a structured set of simple oral instructions, by stepping to the instructed corner in a square. Recently, we demonstrated that one session of QMT can improve cognitive function, including creativity and spatial cognition, in comparison to two alternative training regimens that did not combine motor and cognitive aspects (Ben-Soussan et al., 2013, 2014). In the current study, the QMT is applied for a period of one month, in order to test its efficacy in inducing plasticity.

We have chosen magneto-encephalography (MEG) as the main tool for assessing changes in brain activity, due to its excellent resolution in the temporal domain, as well as its superiority to EEG in terms of effective spatial resolution (Kanda et al., 2000; Genow et al., 2004). In fact, it has been explicitly argued that MEG could be an excellent tool for evaluating the neural correlates of training-induced changes in dyslexia because of its ability to localize the sources of the alpha activation in parallel to the examination of long-distance alpha coherence (Salmelin, 2007). Further theoretical motivation for this choice is provided by the temporal sampling framework (TSF), which has been recently proposed to connect the observed sensorimotor deficits in dyslexia to temporal alterations in neuronal oscillations (Goswami, 2011). We therefore set out to examine the effects of QMT in a group of adult dyslexics and matched controls using MEG alpha power and coherence as electrophysiological dependent measures, as well as reading performance and verbal fluency as cognitive measures.

We hypothesized that dyslexics would show reduced alpha power, altered alpha coherence and lower reading skills at baseline in comparison to controls. We further hypothesized that QMT would increase cerebellar alpha power due to the important role of cerebellar alpha power in voluntary action (Tesche and Karhu, 2000; Ivry et al., 2002). Increased cerebellar alpha power would then serve to normalize alpha coherence (Basar et al., 1997; Andres et al., 1999; Silberstein et al., 2003; Silberstein, 2006; Güntekin and Basar, 2010) and improve reading (Goswami, 2011). We therefore tested whether a 4-week period of daily QMT would: (a) Enhance alpha power and normalize alpha coherence in dyslexic adults; (b) Enhance reading performance. Finally, we tested whether changes in alpha power and inter-hemispheric alpha coherence would correlate with behavioral changes in reading.

## Methods

### Participants

Twenty-two native Hebrew speakers participated in the study: 12 dyslexic participants (5 females and 7 males; mean age = 30 (±6); years of education = 15 (±1)) and 10 controls (7 females and 3 males; mean age = 27 (±5); years of education = 14(±2)). We recruited volunteers who had been previously diagnosed as dyslexic by a clinical or educational psychologist and had a documented history of reading and spelling difficulties. We excluded participants who were further diagnosed with comorbid disorders, including Attention Deficit Hyperactivity Disorder (ADHD), Attention Deficit Disorder (ADD) and developmental coordination disorder (Ramus et al., 2003). All participants provided written informed consent to take part in the study.

### Procedure

**Figure 1:**
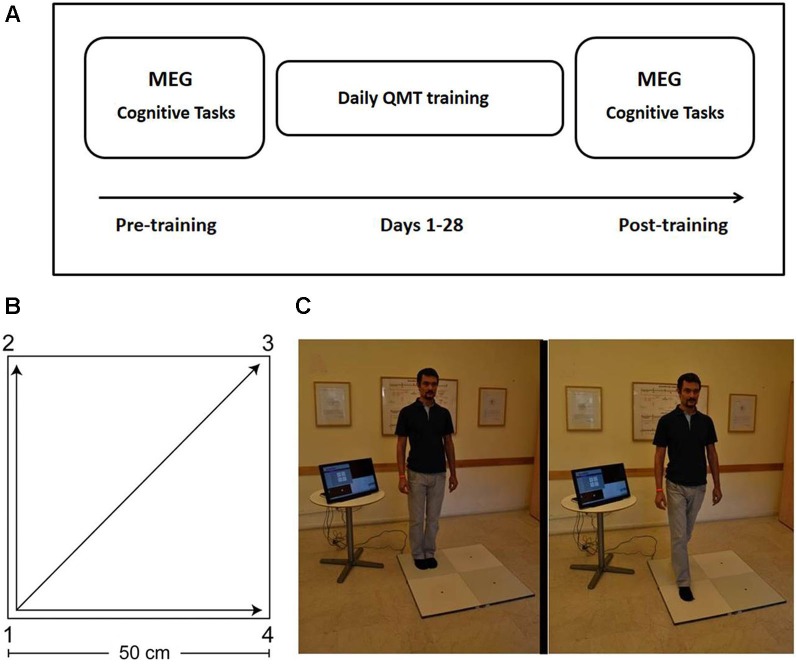
**Study design and Quadrato Motor Training. (A)** Experimental protocol. MEG and behavioral measurements were conducted before and after 4 weeks of QMT training (see text for details). Two different versions of the cognitive tests were presented pre- and post-training in a counterbalanced order. **(B)** The spatial layout of the training space. **(C)** Practice setup. The trainee listens to recorded instructions and takes a step towards the target point. Figure adapted from Ben-Soussan et al. (2014). Quadrato Motor Training = QMT; Magnetoencephalography = MEG.

The study included three phases: pre-training assessment, QMT training, and post-training assessment (See Figure 1). The pre-training session (Day 1) included the following components in this fixed order: (a) Cognitive testing (about 30 min, see Section Cognitive Tasks below); (b) MEG measurements (about 15 min, see Section MEG Data Acquisition below); and (c) QMT training (about 7 min, see Section Quadrato Motor Training below). Post-training assessment took place at the lab on Day 29, and included the cognitive and MEG components as on Day 1 in the same order using matched versions of the cognitive tasks (see Section Cognitive Tasks). Due to technical problems, one dyslexic participant did not complete the behavioral tasks; additionally, the behavioral data for the post-training verbal fluency tasks was incomplete for two dyslexic and one control participants. In those cases where participants failed to complete certain behavioral tests, their MEG measurements were still included in the analysis of the MEG data in order to increase statistical power given the small sample size.

### Quadrato motor training

The participant stood in a quiet room at one corner of a 0.5 m × 0.5 m square and made movements in response to verbal instructions given by an audio tape recording. Participants were instructed to keep the eyes focused straight ahead and their hands loose at the side of the body. They were also told to immediately continue with the next instruction and not to stop due to mistakes. At each corner, there are three possible directions to move. The training thus consists of 12 possible movements (Figure 1). The daily training consisted of a sequence of 69 commands, lasting 7 min. Two variables that were addressed in other studies of motor learning are limb velocity and the decision regarding the responding limb (Criscimagna-Hemminger et al., 2002; Donchin et al., 2003). In order to control these parameters, we used a movement-sequence paced at a rate of an average of 0.5 Hz (similar to a slow walking rate), and we instructed the participants to begin all movements with the leg closest to the center of the square. Starting on day 2, daily QMT sessions were conducted by the participants at home. Home training lasted 27 consecutive days (from Day 2 to Day 28), and lasted 7 min each day.

### Cognitive tasks

The cognitive tasks were performed before the MEG measurement, and lasted for about 30 min. The order of tasks was fixed, starting with the reading test, category-based fluency and then letter-based fluency task. Each task had two different versions, and each of these versions was assigned to the pre- or post-training session in a counterbalanced manner across subjects.

#### Reading test

This test examines single-word reading speed and accuracy. A list of forty five written Hebrew words of increasing difficulty was presented and participants were asked to accurately read as many words as possible from the list in 1 min. The level of difficulty of the words was controlled in terms of word length and number of syllables. In order to minimize learning effects from the pre-test to the post-test, two non-overlapping word lists were created. Each list was presented either before or after training, in a counterbalanced manner. The two lists of 45 words were sampled from a database of rated Hebrew words (Levy-Drori and Henik, 2006), and were matched item-by-item for concreteness, availability of context, familiarity, number of letters and number of syllables. Since several participants from the control group finished the list of words in less than 1 min, the final score represents the number of words which were read correctly in the first 30 s.

#### Category-based fluency task

Participants were asked to say in 1 min as many words as possible belonging to a given semantic category. Two semantic categories were used alternately: (a) Animals; (b) Fruits and vegetables. One category was presented in the pre-training session (Day 1) and the other in the post-training session (Day 29), and the order of the categories was counterbalanced across subjects. Fruits and vegetables were treated as one category in order to avoid the ambiguity between botanical definitions and common usage (as in “avocado”). These categories were chosen because they have comparable norms, and in order to avoid test-retest influence by repeating the same category (Kavé, 2005).

#### Letter-based fluency task

Participants were asked to say in one minute as many words as possible that start with a given letter. We used two Hebrew letters: Bet (/b/) and Gimel (/g/). One letter served for pre-training and the other for post-training, and the order of the categories was counterbalanced across subjects. These letters were chosen because they have comparable norms, and in order to avoid test-retest influence by repeating the same letter (Kavé, 2005).

### MEG data acquisition

Power and coherence measures were collected using the MEG at the beginning and at the end of the month, after performing the cognitive tasks. MEG recordings were conducted with a whole-head 248-channel magnetometer array (4-D Neuroimaging, Magnes 3600 WH) in a magnetically shielded room. During the Rest condition, the participants were asked to refrain from moving and from falling asleep. In addition, the participants were asked to keep their eyes closed, in order to reduce ocular artifacts in the measured signals and to facilitate the localization of potential generator regions of the alpha resting–state oscillations (Goldman et al., 2002). Data acquisition took 15 min. We also collected MEG data using two active tasks which will be reported elsewhere. Before acquiring the data, the head-shape of each subject was digitized. Reference coils located approximately 30 cm above the head oriented by the *x*, *y* and *z* axes were used to record environmental noise. Three accelerometers, one for each axis, attached to the MEG gantry were used to record building vibrations in order to remove artifacts caused by them. The data were digitized at a sampling rate of 1017.25 Hz, and a 0.1 to 400 Hz band-pass filter was used online. The 50 Hz line power fluctuations were recorded directly from the power-line in order to remove the artifact on the MEG sensors.

### Preprocessing MEG data

Power line, heartbeat, and vibration artifacts were removed automatically (Tal and Abeles, 2013). The data were then divided into 1 s epochs. Muscle artifact was estimated by examining the absolute value of all the MEG channels for every epoch, after applying a 20 Hz high-pass filter. For each epoch, the mean absolute value was computed. These values were then converted to z-scores, and epochs with *z*-scores greater than 3 standard deviations were rejected. Eye blinks were not considered as possible artifact for the alpha power processing, because the participants had their eyes closed and did not blink. Some eye movement artifact was still present in the data, but this was in a lower frequency range and was negligible in the alpha frequency range. Two of the 248 channels were noisy (one of the channels registered a constant zero value and the other exceeded 1 nanotesla); these channels were therefore excluded from all sensor and source level analysis. For left-right coherence computation, their homolog channels were omitted as well.

### Source localization

Source localization was applied for the alpha (7–13 Hz) frequency band. Synthetic Aperture Magnetometry (SAM) beamforming (Robinson and Vrba, 1999) was used with multiple spheres forward solution based on the digitized headshape. The neural activity was estimated for a grid of points covering the volume of the brain with 5 mm intervals. The power of activity was calculated for every grid point and for every epoch. Since raw beamforming results are biased toward deep sources it was necessary to normalize the images in order to keep the noise level equal throughout the whole volume of the brain. For this purpose, a pseudo-*z* score was calculated by averaging the power of every location, across epochs, divided by its noise estimate. The noise estimate was determined by the weights (the spatial filter). Deep sources generally have weights with higher values and are therefore noisier. Dividing the power of activity by the square of the weight norm can compensate for this bias (see Equation 3 in Sekihara et al., 2004). The absolute value of the weights of a particular location serves as a noise estimate. The resulting images represent the increase of alpha compared to noise, without being biased to deep sources. The pseudo-*z* value for each location was visualized as the color of voxels in the resulting functional images. The images were transformed to Talairach space by fitting a template MRI to the individual headshapes using SPM8 (Friston et al., 2007) and FieldTrip (Open Source Software for Advanced Analysis of MEG, Oostenveld et al., 2011) packages used with Matlab® R2010b. In order to control for multiple comparisons, a simulation was applied using an Analysis of Functional NeuroImages (AFNI) function (AlphaSim) which determines the probability to get significant clusters of different sizes at random. According to the simulation, at current parameters (given the template brain and spatial resolution used), clusters of voxels with a *p*-value smaller than 0.05 and exceeding one cubic cm (8 voxels) do not count as random noise. We decided to be even more conservative and to take only clusters containing more than 20 voxels at a threshold of *p* < 0.005 (Bunge et al., 2001).

### Coherence

The coherence between left and right sensors was computed using FieldTrip. The data were first baseline-corrected by subtracting the mean of every epoch from the MEG traces. Eighteen channels located along the midline of the helmet were omitted from the coherence analysis, because these channels are likely to present with high coherence based on proximity, since spatially close sensors are likely to pick up very similar activities (Lehnertz et al., 2014).

Fourier transform was then computed using a spectral smoothing box of 1 Hz (meaning that the 10 Hz bin includes 9 to 11 Hz oscillations). The frequencies per time window were computed using a DPSS bell-shaped window. The resulting complex spectrum was used to assess coherence between each channel and its homolog. The coherence value of the 18 channels located along the mid-line of the helmet was set to one, representing perfect coherence between each channel and itself (the vertical red line in Figure 4). The coherence was projected onto a two-dimensional map of the sensor array, using the same left-right coherence value for the left as well as the right sensor, thus creating symmetrical maps of coherence. After the creation of the maps, channels *close* to the midline were excluded from the statistical analysis (in addition to the midline channels). This step was necessary because channels near the midline had high coherence values which did not represent cortical coherence, but simply the fact that the activity of one brain region was measured by two nearby channels. The channel pairs chosen were at least 11 cm apart in order to avoid “false coherence” resulting from close channels that pick up the same source and covered the lateral area of increased coherence (greater than 0.3; Figure 4). This procedure resulted in 59 channel pairs for which left-right coherence was statistically evaluated. Usually when studying inter-hemispheric differences, coherence is computed for one artifact-free channel for each region (e.g., from frontal, central, parietal, and temporal regions) and is computed for channels located in the corresponding regions of the two hemispheres (Osipova et al., 2003; Kikuchi et al., 2011). In fact, the number of chosen pairs is conventionally determined by *apriori* assumptions and therefore restricted to particular regions of interest. However, due to the exploratory nature of the current study, it was important to expand the search across multiple sensors.

**Figure 2:**
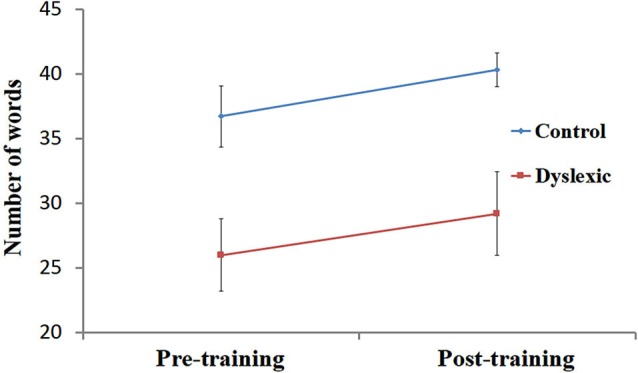
**Reading performance pre- and post-training**. Number of words read correctly (mean ± SEM) in 30 s, pre- and post-training in the dyslexic (red) and control (blue) groups.

**Figure 3:**
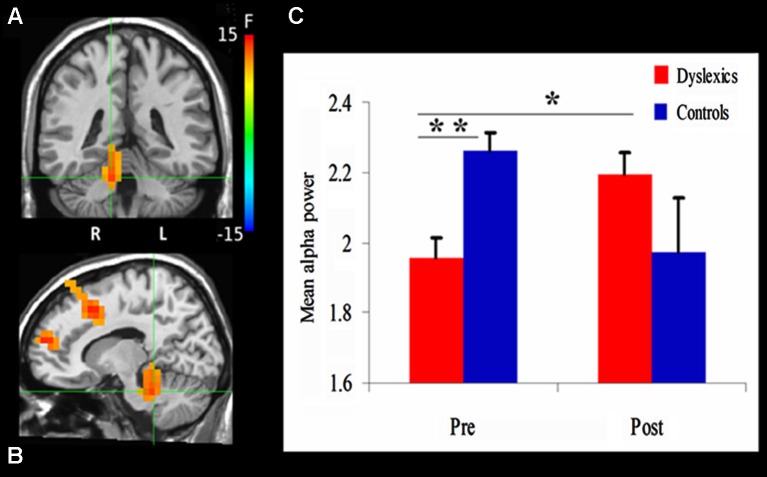
**Changes in alpha power. (A)** and **(B)** demonstrate the significant clusters resulting from the Group (dyslexics, controls) by Training (pre-training, post-training) interaction. Voxels are colored by the *F* statistics, overlaid on coronal **(A)** and sagittal **(B)** views. The statistical map is thresholded at *p* < 0.0025 in addition to a cluster size threshold of 20 voxels. The focus point (green cross) is positioned in the right culmen (Talairach coordinate: 12, −37, −22). **(C)** The bar graph shows alpha power as a function of Group and Training (mean + SEM). * *p* = 0.01; ** *p* = 0.001.

**Figure 4:**
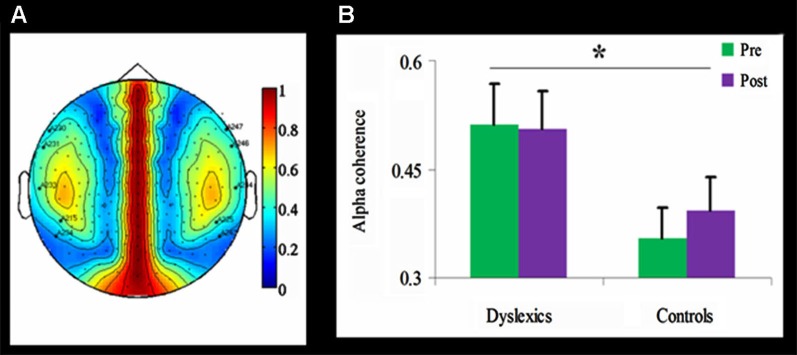
**Alpha coherence in the dyslexic and control groups**.≢wline **(A)** Group differences in inter-hemispheric alpha coherence. The coherence was higher over temporal channels in the dyslexic group compared with the control group (* *p* < 0.01, uncorrected). **(B)** Temporal alpha coherence as a function of Group and Training (mean + SEM), demonstrating significant group differences between the dyslexic and control groups (* *p* < 0.01, uncorrected), as well as a null effect of the training in the dyslexic group and a trend toward an increase in alpha coherence in the control group.

### Statistical analysis

Mixed design ANOVA was used to test the effects of QMT on performance in the reading and verbal fluency tasks, with Training (pre-training, post-training) as a within-subject factor and Group (dyslexic, control) as a between-subjects factor. Statistical parametric maps were produced from MEG data using the AFNI package (Cox, 2012). Mixed design ANOVA was used to test the effects of QMT on alpha activity, i.e., alpha power and alpha coherence. Pearson correlation was used to test the association between behavioral and neuronal changes. The correlation threshold was *p* < 0.05. *Post hoc* comparisons were conducted using *t*-tests.

## Results

### Cognitive results

#### Reading task

Performance on the speeded reading task was entered into a 2 (Group) by 2 (Training) ANOVA. First, a significant main effect for Group [*F*_(1, 19)_ = 7.80, *p* < 0.05] was observed, indicating that, across both time points, the number of words correctly read by the controls (*M* = 38.5, SD = 6.3) was higher than the number of words correctly read by the dyslexic participants (*M* = 27.6, SD = 10.7). Secondly, the analysis yielded a main effect for Training [*F*_(1, 19)_ = 6.89, *p* < 0.05], showing that QMT improved single-word reading performance across both groups (see Figure 2). Finally, the interaction between Training and Group was not significant (*p* > 0.9).

#### Verbal fluency

We conducted two separate analyses for the category-based and letter-based fluency tasks using a 2 (Group) by 2 (Training) ANOVA. No significant main effects or interactions were found, for either the category-based or the letter-based fluency task. The mean scores (i.e., number of words generated in 1 min) of the control participants for the category-based or letter-based fluency were similar to the norms (Kavé, 2005).

Based on previous studies demonstrating significant differences in phonological fluency between dyslexic and controls (Rack et al., 1992; Reid et al., 2007), we conducted a planned comparison between the groups for letter-based fluency, separately for pre- and post-QMT. While a marginally significant difference in phonological fluency was found pre-training between the dyslexic and control groups [*t*_(19)_ = 2.08, *p* = 0.051], no such difference was found following the training. In addition, no differences were found between the groups in semantic fluency, neither before nor after training. See Table 1.

**Table 1:** **Mean scores of letter-based and category-based fluency tasks as a function group and training**.

		**Letter**				**Category**		
	Pre		Post		Pre		Post	
	Mean	SD	Mean	SD	Mean	SD	Mean	SD
Dyslexic	8.7	2.4	9.0	1.9	23.9	5.2	24.9	4.3
Control	10.9	3.1	10.4	2.1	23.8	5.1	25.6	4.0

### MEG results

#### Between-group differences in cerebellar alpha power

We first examined the effect of QMT on alpha power using a mixed design ANOVA, with Training as a within-subject factor and Group (Dyslexia, Control) as a between-subjects factor, for each voxel. The ANOVA revealed a significant Group × Training interaction in a cluster in the right cerebellum. The center of mass of this cluster was located in the right culmen (Talairach coordinates (in mm): 12, −37, −22; *F*_(1,20)_ = 13.3, *p* < 0.0025; See Figure 3A). Before training, cerebellar alpha power was significantly lower in the dyslexic group compared to the control group (*t*_(20)_ = 3.88, *p* = 0.001). Following 4 weeks of daily QMT, cerebellar alpha power significantly increased in the dyslexic group (*t*_(11)_ = 3.08, *p* = 0.01) in contrast to the control group which showed no significant change following training (see Figure 3B).

The ANOVA also revealed a significant Group × Training interaction for three frontal clusters, located in the right superior frontal gyrus (SFG) (Talairach coordinates in (mm): 23, 53, 17) [*F*_(1,20)_ = 16.08, *p* < 0.001], supplementary motor area (SMA) (Talairach coordinates in (mm): 13, 18, 42) [*F*_(1,20)_ = 22.41, *p* < 0.001] and the left middle frontal gyrus (Talairach coordinates in (mm): −27, 8, 57) [*F*_(1,20)_ = 16.01, *p* < 0.001]. While there were no significant differences between the groups in these areas prior to training, the control group showed a significant decrease in alpha power in the left medial frontal gyrus (MFG) (*t*_(9)_ = 4.54, *p* < 0.005), right SFG (*t*_(9)_ = 3.73, *p* < 0.005) and SMA (*t*_(9)_ = 3.69, *p* < 0.005) following 4 weeks of daily QMT. On the other hand, the opposite pattern was observed in the dyslexic group in which alpha power increased in the right SFG (*t*_(11)_ = 2.66, *p* < 0.05).

#### Coherence

Inter-hemispheric alpha coherence was tested using a mixed design 2-way ANOVA for each of the 59 channels, with Training as within-subject factor and Group as between-subjects factor. Across both time points, inter-hemispheric alpha coherence was significantly higher in the dyslexic group compared to the control group for five channel pairs (*F*_(1,21)_ > 8.35, *p* < 0.01, uncorrected; See Figure 4). No main effect for Training or interaction was found.

#### Neuro-cognitive correlations

**Figure 5:**
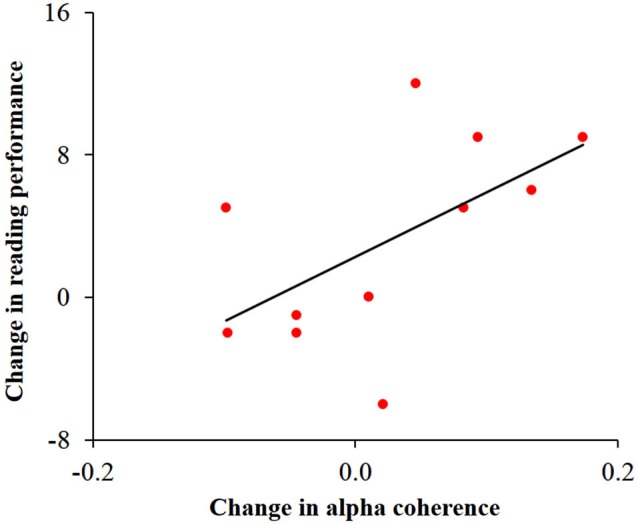
**Correlation between change in temporal alpha coherence and change in reading performance in the dyslexic group**. Change in alpha coherence, calculated by the subtraction of pre- from post-training, was positively correlated with the change in number of words read correctly in 30 s (*r* = 0.58, *p* < 0.05).

In order to study the possible associations between change in alpha activity and change in reading performance, we calculated Pearson correlations between behavioral and neuronal change within each group. This analysis was motivated by previous studies relating reading, cerebellar activity and alpha coherence (Nicolson et al., 2001; Weiss and Mueller, 2003; Arns et al., 2007). Change in speeded reading was calculated as the difference between the number of words read correctly in 30 s before and after training. Change in cerebellar alpha power was calculated as the difference between pre- and post- training cerebellar alpha power of the cluster which was found to have the significant Group × Training interaction. Change in inter-hemispheric alpha coherence was calculated as the difference between pre- and post-training values of the bilateral temporal alpha coherence. No significant correlation was found between change in cerebellar alpha power and change in reading in the two groups. Yet, as can be seen in Figure 5, change in temporal alpha coherence was positively correlated with the change in reading score (*r* = 0.58, *p* < 0.05, *n* = 11; uncorrected) in the dyslexic group but not in the control group. Using the Fisher *r*-to-*z* transformation, we calculated the *z* value to assess the significance of the difference between two correlation coefficients. The results indicated a significant difference between the two correlation values (*z* = 1.87, *p* < 0.05).

## Discussion

Our results contribute two novel findings with regards to the cerebellar involvement in dyslexia: First, we show that cerebellar alpha activity prior to training is lower in dyslexics compared to controls. Second, a 4-week training program enhanced cerebellar alpha activity in dyslexics, but not in controls. Two other important findings are reported here for the first time: First, QMT over a period of 4 weeks improves reading speed in adults, and second, the improvement in reading performance is associated with increase in temporal alpha coherence in the dyslexic group. Below, we discuss these results in the context of different approaches to dyslexia and examine the possible role of the cerebellum in this neurodevelopmental disorder.

### QMT enhances performance in a speeded reading task

This study was inspired by the controversial body of research that examines the connection between reading and the sensorimotor systems (e.g., Flöel et al., 2003; Pulvermüller, 2005), in the context of novel discoveries about the benefit of daily sensorimotor practice for cognition (Ben-Soussan et al., 2013). We found improved reading skills in both groups as a result of 4-week QMT. Commonly, experimental studies attempt to enhance reading abilities and phonological functions by providing a training program for the target skill. Here we show that sensorimotor training might be beneficial for improving reading skills even though the practice relies on faculties that are not directly related to reading. Indeed, the QMT is based on a series of motor responses to verbal commands, which involve functions such as spatial cognition and response inhibition (Ben-Soussan et al., 2014). QMT-related improvement in the speeded reading task was found using different stimulus lists at each time point (pre- and post-training) and is thus considered to result from QMT and not from test-retest effects. This finding is also in agreement with previous results showing cognitive improvement in non-dyslexics adults even following short term QMT (Ben-Soussan et al., 2013, 2014).

In addition to group differences in reading score measured at baseline, a trend of a lower score in the phonological fluency task was observed pre-training in dyslexics compared to controls. This trend was not observed following 4 weeks of daily QMT, suggesting that QMT helped in normalizing the performance on this task. Again, the use of different categories at each time point (pre- and post-training) provided support to the interpretation that it is probably the QMT that normalized performance and not the repetition of the task. In addition, no differences were found between the groups in semantic fluency, neither before nor after training. This supports the view that dyslexia is more related to phonological than to semantic impairment (Leggio et al., 2000).

### QMT enhances cerebellar alpha power in dyslexic adults

In line with our hypothesis, cerebellar alpha power was significantly lower in the dyslexic group prior to training in comparison to the control group (see Figure 3C). Importantly, following 4 weeks of daily QMT, we found that cerebellar alpha significantly increased in the dyslexic group in the right culmen, a region which has been previously reported to be related to language processing (Luke et al., 2002; Pernet et al., 2009a,b; Rudner et al., 2013). This finding may reflect the role of the cerebellum as a general timing mechanism for both sensorimotor and cognitive processes (Ivry, 1997; Tesche and Karhu, 2000; Ivry et al., 2002; Tesche et al., 2007), such as the acquisition of sensorimotor skills and response readiness (Martin et al., 2006). It might also be linked to the critical involvement of the cerebellum in the coordination of smooth movements, maintenance of balance and posture, visually guided movements and motor learning (for review see Manto et al., 2012), which are inherent components of the QMT.

In addition to cerebellar changes, we found differences between dyslexics and controls in frontal alpha activity following 4 weeks of daily QMT. While alpha power in the left MFG and SMA significantly decreased in the control group, the dyslexic group showed increased alpha power in right SFG. These regions have been previously reported to be related to movement and language processing (Binder et al., 1997; Eckert et al., 2003; Neumann et al., 2004). Contrary to the dyslexics, the control group showed a trend towards a reduction in cerebellar alpha power; however, there was a notable increase of the control group’s dispersion around the mean, which might account for the lack of statistical significance of this effect. In line with previous findings on motor practice, it is possible that the reduction of frontal and cerebellar activity indicates that practice became simpler as control of movement and coordination improve (Lacourse et al., 2004).

Previous work based on one session of training reported decreased frontal alpha activity in healthy young subjects (Ben-Soussan et al., 2013). This decreased frontal activity was mostly observed following simple motor training, indicating that changes in these regions might be related to motor learning as well as action observation and intention understanding (Exner et al., 2002; Dapretto et al., 2005). These results are compatible with Goldberg et al. (2006) who reported a complete segregation between self-related and sensorimotor activity in relevant cortical regions using functional neuroimaging. Their results showed that frontal regions were functionally inactive during sensorimotor tasks and active during self-engaged tasks. It is therefore possible that reduced activity of frontal regions at rest in controls signifies automaticity of sensorimotor components as a result of the repetition of the same sequence of QMT for 4 weeks.

### Alpha coherence in dyslexic adults compared to controls

Alpha coherence is important for cognitive and sensory processing (Weiss and Mueller, 2003; Ben-Soussan et al., 2013). Previously, EEG studies revealed increased coherence in dyslexic children, especially between temporal areas during rest (Shiota et al., 2000; Arns et al., 2007). Contrary to prior results showing increased inter-hemispheric alpha coherence following a single session of QMT (Ben-Soussan et al., 2013), in the current study neither group showed a significant increase in alpha coherence following one month of daily QMT. It should be noted that the previous results were obtained using EEG and not MEG. In fact, calculating connectivity from sensor level recordings is not straightforward, as these recordings are highly dependent on the effects of field spread. In other words, coherence measured by MEG reflects fewer sources because the spatial scale of the MEG sensors is smaller resulting in inflated estimates. Moreover, EEG and MEG are different in their sensitivity to radial and tangential dipoles (Srinivasan et al., 2007). This points to the necessity to integrate different methods in the study of training-induced plasticity.

Importantly, in the current study coherence analysis confirmed increased inter-hemispheric alpha coherence in the dyslexic group compared to the control group across time points. The increased inter-hemispheric coherence, especially between the temporal areas, may reflect the connection between left and right superior temporal sulci, which are considered to be necessary for phonological processing (Hickok and Poeppel, 2004). These findings converge with independent data from diffusion imaging showing that children with lower phonological and reading skills have higher anisotropy in temporal-callosal fiber tracts (Ben-Shachar et al., 2007; Dougherty et al., 2007).

Consequently, we propose that the increased coherence found in the dyslexic group may reflect a compensation mechanism (Roberts and Kraft, 1989; Arns et al., 2007). This suggestion further accords with the view that both left and right posterior superior temporal cortices are required for phonological processing (Hickok and Poeppel, 2007). Indeed, earlier models of dyslexia promoted the premise that complex cognitive functions, such as the translation of graphic symbols into a phonemic code, depend on component processes from both cerebral hemispheres, and that at least some subtypes of dyslexia may be due to abnormal inter-hemispheric communication (Gazzaniga, 1973; Gladstone and Best, 1985; Wolff et al., 1990). The association between change in coherence and reading performance revealed a significant positive correlation only in the dyslexic group, suggesting that the underlying mechanisms of improved reading observed in this study are connected with increased inter-hemispheric communication in the alpha range. Due to the low power of the correlation analysis (*N* = 11) and the non-significant effect of training on alpha coherence, this finding should be treated as suggestive, and should be tested in future larger MEG studies of developmental dyslexia. Nonetheless, the positive correlation reveals that participants who showed higher improvement of speeded reading also demonstrated increased bilateral temporal alpha coherence, in addition to the general increase in coherence observed in dyslexia. Some researchers aimed at normalizing brain activity (and consequently ameliorating behavioral and cognitive deficits) in various developmental disorders by suppressing hyper-connectivity (Pineda et al., 2012). Similarly, in stroke rehabilitation, applying brain stimulation to inhibit inappropriate activity of non-specialized areas has been argued to offer an effective avenue of treatment (Naeser et al., 2005). However, ameliorating cognitive deficits in developmental disorders may not necessarily be achieved through suppressing abnormal connectivity, because the observed hyper-connectivity does not necessarily reflect a dysfunction (Arns et al., 2007). Indeed, findings from neurofeedback training show that, contrary to the expected effects, 6 months of training induced an increase in alpha coherence, which might be related to improved attention (Breteler et al., 2010).

### Towards a new approach to understanding and treating dyslexia

Existing methods of treating dyslexia usually rely on phonetic and reading materials which aim at dealing directly with the linguistic impairments. Nevertheless, dyslexia, as well as other developmental disorders, should not be interpreted as being impairments in a single cognitive process (Castles and Coltheart, 1993; Pernet et al., 2009a). These cognitive impairments should rather be regarded as the endpoint of an abnormal developmental process, reflecting the interactions of multiple potentially deficient processes as well as compensatory processes (Thomas and Karmiloff-Smith, 2002). The current study attempted to investigate dyslexia-related differences in specific regions, in inter-hemispheric coherence, and in response to intervention. In this way, the differences between groups in the training-induced electrophysiological effects may provide further insight into the deficient and compensatory processes that characterize dyslexia.

In line with our results, we suggest that both the deficient cerebellar alpha power and possibly compensatory alpha coherence may be connected to the cerebellum’s role as a generator of alpha activity, and that sensorimotor training may lead to cerebellar plasticity which could eventually rebalance the system. In this respect, altered cerebellar oscillatory activity may be the source of the deficit in dyslexia since it could be viewed as a neural system that mediates cortical communication (Andres et al., 1999; Silberstein et al., 2003). Our preliminary findings also disclose that sensorimotor training can be a practical intervention in dyslexia because of its potential to facilitate cerebellar oscillatory activity. Exploring how neuronal oscillation and cerebellar function change as a result of training may have valuable implications for educational neuroscience.

### Limitations

The current study is a preliminary attempt to examine empirically the question of system modulation, which is required for improving reading in dyslexia. The main limitations of the current study are the small sample size and the use of only one training paradigm. The choice of QMT was made based on previous studies in which it was demonstrated that cognitive changes, namely increased creativity and improved spatial cognition, are QMT specific, and are not observed in two control groups (Ben-Soussan et al., 2013, 2014). In the future, a study on a larger sample that includes several training regimes may extend the current results. In future research, it would be important to include a passive control group; in particular, a dyslexic passive control group would ensure that any test-retest effects that were controlled for with normal-readers and with the two different versions of the tasks are not different in participants with dyslexia.

So far, EEG studies have generally avoided studying cerebellar function because of the complex folding of the cerebellar cortex. As for MEG, signals can be obtained from the cerebellum especially within the alpha range (Ivry, 2000; Park et al., 2011). However, source localization makes it difficult to distinguish between signals arising in the cerebellar cortex and deep nuclei. Thus, our results should be interpreted keeping these limitations in mind. Regarding the coherence analysis, the statistical significance was not corrected for 59 comparisons and should be therefore evaluated with caution. However, since no previous study has shown similar left-right coherence effects it was impossible for us to focus on channels of interest and reduce the number of multiple comparisons. The results we report here can therefore be considered as exploratory, and should be confirmed in future studies. We expect that future studies will utilize independent imaging methods in order to examine the role of the cerebellum in reading and dyslexia, and the impact of QMT on cerebellar activity and connectivity.

## Conclusions

The current MEG study is in line with previous studies suggesting that dyslexia may be related to cerebellar dysfunction. Four weeks of daily QMT enhances reading performance and cerebellar alpha oscillations in dyslexic participants. In addition, improved reading performance in dyslexics correlates with inter-hemispheric temporal alpha coherence. Our results suggest that cerebellar impairment in dyslexia can be modulated by sensorimotor training. Most importantly, the investigation of training-induced effects on reading performance provides a unique opportunity to gain insight into the relation between behavioral and neuronal changes. A better understanding of the functional coordination between cortical regions and the cerebellum would be necessary to pinpoint some of the underlying sources of dyslexia and to create training paradigms for clinical purposes. The current study provides an important step in bringing together different approaches to study the sources and treatment of dyslexia and the scientific value of sensorimotor training.
